# CACGAACTACCCTAA

**DOI:** 10.15252/embr.202254646

**Published:** 2022-01-26

**Authors:** Howy Jacobs

**Affiliations:** ^1^ Tampere University Tampere Finland; ^2^ La Trobe University Melbourne Australia

**Keywords:** Evolution & Ecology, History & Philosophy of Science

## Abstract

Will future archeaologists and anthropologists be able to make sense of what happened to our species in the 21st century?

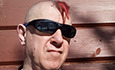

The discovery of a primitive stone carving bearing the simple inscription “CACGAACTACCCTAA” has greatly excited the world of anthropogenetics and resuscitated debate on the origins of modern humans.

Estimated to be at least 1.5 million years old, and thus dating to near the end of the Applocene era, the carving reveals three extraordinary facets of human evolution: first, that our primitive ancestors, somewhat ironically described as *Homo sapiens* (though many authorities still prefer to use the earlier term *Homo perniciosus*), had developed primitive writing skills and therefore some kind of language before they suddenly vanished from the fossil record. Even though it seems premature to regard the use of 4 different symbols as a genuine alphabet, it does suggest that they had achieved at least the first step toward what we would today recognize as a society.

Other experts have suggested that the four symbols might have a religious significance, possibly corresponding with the four major planets visible to the naked eye. However, the sequence of symbols does not appear to correspond with any known planetary event or phenomenon, and it is extremely doubtful that *H. sapiens* was capable of the analytical skills that we nowadays would group under the term “mathematics”. Could it be a fragment from the tombstone of a great warrior king? Maybe CACGAACTACCCTAA, however it was vocalized, was his name. Either way, the discovery, alongside several fossilized fragments of bone, allows the species of origin to be unambiguously assigned.

Second, although the exact method used to produce the carving is unknown, it indisputably reveals that its creators had developed tools of some kind, presumably harder rocks that were fashioned into primitive chisels or awls. This adds to the mystery of the species’ disappearance, given that our own, more recent ancestors moved on very rapidly from the Stone Age to the information age. Why did *H. sapiens* become extinct, never making any progress beyond sharpened rocks, whereas *H. methodicus* created the rich cultures and technological achievements of today’s world in less than 5,000 years?

The third and perhaps most extraordinary aspect of the discovery is that the artifact was dug out of one of the moraines at the southern edge of the barren, frozen wasteland that is the continent of North America. It supports the hitherto ridiculed hypothesis that a brief interglacial warming period occurred near the end of the Applocene, during which our almost‐human ancestors were present very far away from our natural home in Africa. How or why they went there is an enduring mystery, and it raises the intriguing question of what else lies buried in those northern ice‐caps? There is clearly more to *Homo sapiens* than we have hitherto realized.

From the few other surviving skeletal remains of the period, we know that other hominids and ape‐like creatures co‐existed with *H. sapiens*, during at least a part of their evolution. *H. sapiens* appears to have overlapped with several far more advanced species, such as *H. neanderthalis*. *H. sapiens* has even been suggested by some paleo‐anthropologists to have interbred with them. But those more sophisticated human ancestors also became extinct, more or less at the same time (give or take 30,000 years or so), perhaps at the hand of their more acquisitive and aggressive congeners. Or were they victims of the same climate catastrophe?

The world’s latest and most powerful Artificial Intelligence system (ArtFul) has concluded, enigmatically, that the inscription was a “desperate cry for help from a dying civilization”. Since, as yet, we barely understand how ArtFul works, we cannot fathom the basis of this deduction.

